# Molecular characterisation and additional morphological descriptions of *Eimeria* spp. (Apicomplexa: Eimeriidae) from brown kiwi (*Apteryx mantelli* Bartlett)

**DOI:** 10.1007/s11230-023-10086-4

**Published:** 2023-02-24

**Authors:** Sarah M. Coker, Kate McInnes, Emilie Vallee, Patrick Biggs, William E. Pomroy, Laryssa Howe, Kerri J. Morgan

**Affiliations:** 1 School of Veterinary Science, Massey University, Private Bag 11 222, Palmerston North, New Zealand; 2 Wildbase, Massey University, Private Bag 11 222, Palmerston North, New Zealand; 3 Department of Conservation, PO Box 10-420, Wellington, 6143 New Zealand; 4 School of Natural Sciences, Massey University, Private Bag 11 222, Palmerston North, New Zealand

## Abstract

Brown kiwi (*Apteryx mantelli* Bartlett), a ratite endemic to New Zealand, is currently listed as “Vulnerable” under the IUCN classification system due to predation by introduced mammals. Operation Nest Egg (ONE) raises chicks and juveniles in predator-proof enclosures until they are large enough to defend themselves. These facilities experience an environmental accumulation of coccidial oocysts, which leads to severe morbidity and mortality of these kiwi. Four species of coccidia have been morphologically described from sporulated oocysts with additional opportunistic descriptions of endogenous stages. This research continues the morphological descriptions of these species of *Eimeria* with an additional novel morphotype also morphologically described. It also provides the first genetic characterisation targeting the mitochondrial cytochrome *c* oxidase I (COI) gene. Based on these findings, it was determined there are at least five morphotypes of *Eimeria* that infect brown kiwi and co-infections are common at the ONE facilities surveyed. The COI amplicon targeted for this study was sufficient to provide differentiation from other members of this genus. Sanger sequencing yielded ambiguous bases, indicating the need for more in-depth sequencing.

## Introduction

Brown kiwi (*Apteryx mantelli* Bartlett), a ratite endemic to New Zealand, are listed as “Vulnerable” under the IUCN classification system (BirdLife International 2017). Predation by introduced mammals, particularly domestic dogs (*Canis lupus familiaris* Linnaeus), stoats (*Mustela erminea* Linnaeus) and rats (*Rattus* spp.), has played a major role in population decline (McLennan et al., 1996; McLennan et al., 2004). To limit predation on juvenile kiwi, the “Operation Nest Egg” (ONE) programme was developed to bring eggs and recently hatched wild chicks into captivity, where they are raised in a predator-free environment until they reach at least 1kg before being released (Colbourne et al., 2005). Unfortunately, due to a build-up of environmentally resilient *Eimeria* spp. oocysts (Apicomplexa: Eimeriidae, Schneider 1875) and increased density of immunologically naïve hosts in Operation Nest Egg (ONE) facilities, the juvenile brown kiwi suffer morbidity and mortality delaying the programme’s ability to release kiwi into the wild (Williams, 2001; Yabsley, 2008; Morgan et al., 2012; Morgan et al., 2013). Although, coccidia infections of kiwi were first reported in the 1970s, in-depth, morphological descriptions of oocysts and endogenous stages were not described until more recently. Morgan et al. (2017) provided descriptions of four species (*Eimeria mantellii*, *E. kiwii*, *E. paraurii*, *E. apteryxii*) from sporulated oocysts shed by six captive brown kiwi from two ONE sites. Other reports of coccidia in kiwi include four unsporulated morphotypes described by Thompson and Wright (1978); three morphologically distinct gametocytes found in the intestinal tract (Morgan et al., 2012); one gametocyte reported in the renal system; and meronts in multiple visceral organs (Morgan et al., 2013). These reports likely overlap, describing stages of the same morphotype; however, these connections require further verification.

Accurate detection of coccidia in individual birds provides vital information regarding treatment and control of disease within kiwi rearing facilities. More detailed identification of coccidia to a species level would enable key insights for management of disease within and between kiwi populations, including issues such as treatment efficacy; translocation of disease between populations; and determination of pathogenicity of individual parasite species (Morgan et al., 2013; Morgan et al., 2014; Taylor et al., 2019). When, for example, a brown kiwi tests positive for a particular species of *Eimeria* that is known to be highly pathogenic, decisions around management can be made. These decisions may include a delay to a planned translocation of that individual as the stress involved with this may increase their chances of experiencing severe clinical disease post-release.

Historically, species of coccidia have been classified only on morphological descriptions and seldomly providing both exogenous and endogenous descriptions (e.g., Medina et al., 2019; McAllister and Hnida, 2020). This methodology is limited by morphological similarities between oocysts of different *Eimeria* spp. and does not provide any information regarding endogenous development and subsequent pathology. In order to definitively connect clinical disease with sporulated morphotypes, experimental infection and pathogenicity trials have been undertaken in domestic host species, such as the Barbary partridge *Alectoris barbara* (Bonnaterre) and chickens (*Gallus gallus domesticus* Linnaeus) (Fernandez-Alvarez et al., 2016; Jenkins et al., 2017). However, this approach is not possible in species such as the brown kiwi, which, from a conservation perspective, are considered to be nationally “At Risk: Declining” (Robertson et al., 2017). The use of molecular tools could be an effective approach to link morphological identification with key questions about the epidemiology, virulence, and pathogenicity of these species. A previous study by Morgan (2013) explored this approach and used Sanger sequencing to target the 18S rRNA gene, and ITS-1 and ITS-2 regions. Whilst the 18S rRNA sequences confirmed the genus, the ITS-1 and -2 regions were found to be extremely variable in length (ITS-1, 195-515bp; ITS-2, 60-714bp). While some variation in target sites is needed to differentiate species, discerning the points of variation that differentiate genetically distinct species of *Eimeria* can be exceedingly difficult especially if the target gene varies greatly within a single species of coccidia. More recently, the successful molecular identification of *Isospora* spp. Schneider 1881 and *Eimeria* spp. using the mitochondrial cyctochrome c oxidase subunit 1 gene indicates that this gene provides higher resolution than 18S rRNA gene and ITS regions for phylogenetic studies (da Silva-Carvalho et al., 2018; McAllister et al., 2019; Woodyard et al., 2019; da Silva-Carvalho et al., 2020; Maronezi et al., 2022) Therefore, the following research presents additional morphological descriptions of oocysts shed by brown kiwi and documents a novel morphotype and provides partial mitochondrial cytochrome *c* oxidase I (COI) genes analysed with Sanger sequencing.

**Table 1:** Brown kiwi (*Apteryx mantelli*) pooled dropping samples used for morphological and molecular characterisation of kiwi *Eimeria* species

Sample	Region	Location	Year	Month Collected	Kiwi Age
K1	Manawatu	Pūkaha	2017	December	adult
K2	Manawatu	Pūkaha	2018	October	juvenille
K3	Manawatu	Nga Manu Nature Reserve	2018	December	Not known
K4	Canterbury	Orana Wildlife Park	2017	August	adult
K5	Bay of Plenty	NKHA	2018	April	Not known
K6*	Bay of Plenty	NKHA	2018	February	juvenille
K7	Waikato	Warrenheip	2017	October	juvenille

## Methods

***Sample collection***

Seven brown kiwi faecal samples from five locations, collected between 2017 and 2018 collected from routine diagnostic and transportation health screenings, were chosen for this study based on a combination of factors including: location, the success of oocyst sporulation, oocyst load (oocysts per gram; OPG), amount of sample remaining, and diversity of coccidial morphotypes (Table 1). All seven samples were pooled droppings that were collected from outdoor enclosures using sanitary, single-use collection bottles. Pūkaha National Wildlife Centre, Mount Bruce, Wairarapa (40°43′35.0″S 175°38′23.6″E; n = 2); The National Kiwi Hatchery Aotearoa, Rotorua (38°06′33.5″S 176°13′14.3″E; n = 2); Nga Manu Nature Reserve, Waikanae (40°51′40″S 175°03′40″E; n=1); and Orana Wildlife Park, Christchurch (43°28′02″S 172°27′40″E; n = 1) house ONE kiwi in enclosures containing one or two individuals at a time prior to release into a crèche. Warrenheip, Cambridge/Waikato (37°56′19.7″S 175°43′08.5″E; n = 1) is a 16-hectare crèche that can accept up to 12 juvenile kiwi at a time. All samples were kept at room temperature and sent to Massey University, Palmerston North, New Zealand within one month of collection. On arrival at Massey University, all dropping samples were assigned a code based on location as well as an identification number that reflects the individual kiwi host or group of hosts, if known. If unknown, a new identification number was assigned to that sample. Repeat sampling from the same host(s) were differentiated by the date of submission. All samples positive for coccidia were processed as described below.

**Table 2:** Primer sets used for amplification of partial COI genes from kiwi coccidia

Gene Target		Primer Name	Primer Sequence	Amp. Size	Reference
**COI**	1′	Cocci_COI_For Cocci_COI_Rev	5′- GGT TCA GGT GTT GGT TGG AC -3′ 5′- AAT CCA ATA ACC GCA CCA AG -3′	~780	Ogedengbe et al. (2011)
	2′	COIF2 COIR2	5′- TAA GTA CAT CCC TAA TGT C -3′ 5′- GTC ATC ATA TGR TGT GCC CA -3′	~465	Yang et al. (2013)

^*^K6 is a sample collected from a bird infected The National Kiwi Hatchery Aotearoa that had been sent to Wildbase Hospital, Massey University, Palmerston North for treatment for high coccidial burdens. Pūkaha refers to Pūkaha National Wildlife Centre; NKHA refers to The National Kiwi Hatchery Aotearoa.

***Oocyst detection, sporulation and storage***

Samples were screened for coccidia using the Mini-FLOTAC technique, as described in Coker et al. (2020). The oocysts were sporulated as described by Duszynski and Wilber (1997). Briefly, small aliquots of droppings were mixed with 2% aqueous (w/v) potassium dichromate (K_2_Cr_2_O_7_) at a minimum 1:5 ratio. After distributing these mixtures thinly over the bottom of Petri dishes (100 mm x 15 mm) to ensure oxygenation, the samples were incubated for 15 days at room temperature. Distilled water was added as needed to keep the contents moist and regularly agitated for gas exchange during the incubation period. Sporulated oocysts were stored at room temperature in 25 ml, sealed flasks.

***Sporulated oocyst isolation and measurement***

One ml aliquots in 1.5 ml microcentrifuge tubes were spun at 18,800 x g for 7 min to form a pellet. The supernatant was removed and replaced with 1 ml of dH_2_O, vortexed, then centrifuged again. This rinsing was repeated 4-6 times until the supernatant was colourless, then 500 $\mu$ l magnesium sulphate solution (MgSO_4_, SG 1.28) was added. The sample was homogenised with a disposable pipette and left to rest for at least 1 min. Drops of this mixture were suspended from the underside of a coverslip that was carefully placed on a cavity slide. The coverslip was temporarily adhered to the cavity ring using water.

Using a light (LEICA DM750) microscope at 1000x with a LEICA ICC50W mounted camera (Leica Microsystems GmbH, Wetzlar, Germany) calibrated with a 1.0mm stage micrometre (Olympus Optical Co. Ltd., Tokyo, Japan), pictures of individual oocysts were taken under oil immersion at multiple focal points. Morphological features were measured using ImageJ, v1.51s (Schneider et al. 2012) and described for up to 84 oocysts following the guidelines provided by Duszynski and Wilber (1997). Sporocysts were only measured if the entire length was in focus. Type imaging was performed on an Olympus IX83 microscope outfitted with DIC optics using a 100x (NA1.4) objective lens. All images were captured with a Retiga 6000 monochrome camera (QImaging) controlled by cellSens Dimension software (v1.18; Olympus) with a 2x2 bin resulting in apparent pixel sizes of 90.8 nm.

***Molecular analysis***

*Controls*

Immucox® Breeders and Layers (Pacificvet, Christchurch, New Zealand) live vaccine (sample IMMU) that contains *Eimeria acervuline* (Tyzzer 1929)*, Eimeria maxima* (Tyzzer 1929)*, Eimeria necatrix* (Johnson 1930)*,* and *Eimeria tenella* (Railliet and Lucet 1894) was used as a positive control for the PCR reactions. Sterile water controls were included as negative controls for the extraction process and PCR reactions.

*Extraction of DNA*

Aliquots (0.15 g) of samples that were included in the morphological descriptions (n = 7) were stored at -80°C for 12-24 hrs; cycled 3x between liquid nitrogen and 100°C for 5 min at each temperature; and incubated overnight with 40 $\mu$ l Proteinase K (ThermoFisher Scientific, Waltham, MA, USA) at 56°C to break open the unsporulated oocyst walls. DNA was extracted using the ZR Quick-DNA Fecal/Soil Microbe DNA Miniprep Kit (Zymo Research, Orange County, CA, USA) according to the manufacturer’s instructions.

*COI DNA amplification*

A nested PCR protocol targeting the mitochondrial cytochrome *c* oxidase I (COI) region was adapted from Yang et al. (2013) and Ogedengbe et al. (2011) (Table 2). The primary amplification was a 50 $\mu$ l reaction with 1 x PCR buffer, 2.5 mM MgCl_2_, 0.2 mM dNTPs, primers Cocci_COI_F and Cocci_COI_Rev each at 1.0 mM, 0.2 mg/mL BSA, and 2 U Platinum *Taq* DNA Polymerase (Invitrogen) with an initial denaturation at 96°C for 5 min; 40 cycles of 94°C for 20 sec, 59°C for 30 sec, and 72°C for 90 sec; and a final extension at 72°C for 10 min. The secondary protocol used a 50 $\mu$ l reaction with 1 x PCR buffer, 2.5 mM MgCl_2_, 0.2 mM dNTPs, 0.2 mM COIF2, 0.2 mM COIR2, 0.2 mg/mL BSA, and 2 U Platinum *Taq* DNA Polymerase (Invitrogen). The secondary conditions had an initial denaturation at 96°C for 5min; 40 cycles of 94°C for 20 sec, 54°C for 30 sec, and 72°C for 90 sec; and a final extension at 72°C for 10 min (Yang et al., 2013).

*Sequencing*

Amplicons were run on a 1.5% w/v agarose gel containing Invitrogen UltraPure Agarose (ThermoFisher Scientific, Waltham, MA, USA) and visualised with RedSafe Nucleic Acid Staining Solution (iNtRON Biotechnology, Gyeonggi-do, South Korea). Samples with bands at the correct size (~465 bp) were purified using PureLink PCR Purification Kit (Invitrogen) and submitted to the Waikato DNA Sequencing Service (The University of Waikato, Hamilton, NZ) for initial Sanger sequencing. If one or both of the reads had an ABI % quality score below 50%, the samples were reamplified and sent to the Massey Genome Service (Massey University, Palmerston North, NZ) for resequencing.

*COI phylogenetic analysis*

The COI sequences obtained in this study and 61 reference sequences (including *Eimeria* Schneider 1875) spp., *Toxoplasma gondii* (Nicolle & Manceaux 1909), *Neospora caninum* (Dubey et al. 1988), *Isospora* sp. (Schneider 1881), and *Hammondia* spp.(Frenkel & Dubey 1975) obtained from the GenBank database were aligned using ClustalW (Thompson et al., 1994) and trimmed to 459 bases (including spaces) using Geneious v11.0.5 (Biomatters, Auckland, New Zealand). This alignment was used to build a maximum likelihood tree in Geneious; the Tamura-Nei 93 distance model with 1000 replicates was used to calculate branch length. *Toxoplasma gondii* served as an outgroup.

## Results

**Morphology**

Overall, 191 individual oocysts from pooled brown kiwi droppings (n = 7) were measured for this study (Table 3). All samples (7/7) contained coinfections, ranging from two to five morphotypes per sample, and all previously described *Eimeria* spp. were identified (Morgan et al., 2017). No single morphotype was associated with a particular sample. The only sample that contained five morphotypes (WP01), included a single oocyst resembling the previously characterised *E. paraurii* (Morgan et al., 2017).

**Table 3:** The number of *Eimeria* oocysts measured from brown kiwi (*Apteryx mantelli*) collected from 2017-2018 in New Zealand

*Eimeria* species	K1	K2	K3	K4	K5	K6	K7	Total
*E. kiwii*	1	–	2	2	16	3	10	34
*E. mantellii*	–	7	1	1	–	2	2	13
*E. apteryxii*	4	35	1	8	–	7	4	59
*E. paraurii*	–	–	–	–	–	–	1	1
E. paopaoii*	1	5	–	4	51	1	22	84
Total	6	47	4	15	67	13	39	191

^*^E. paopaoii is a new fifth morphotype (M5) reported in this study

**Fig. 1:**
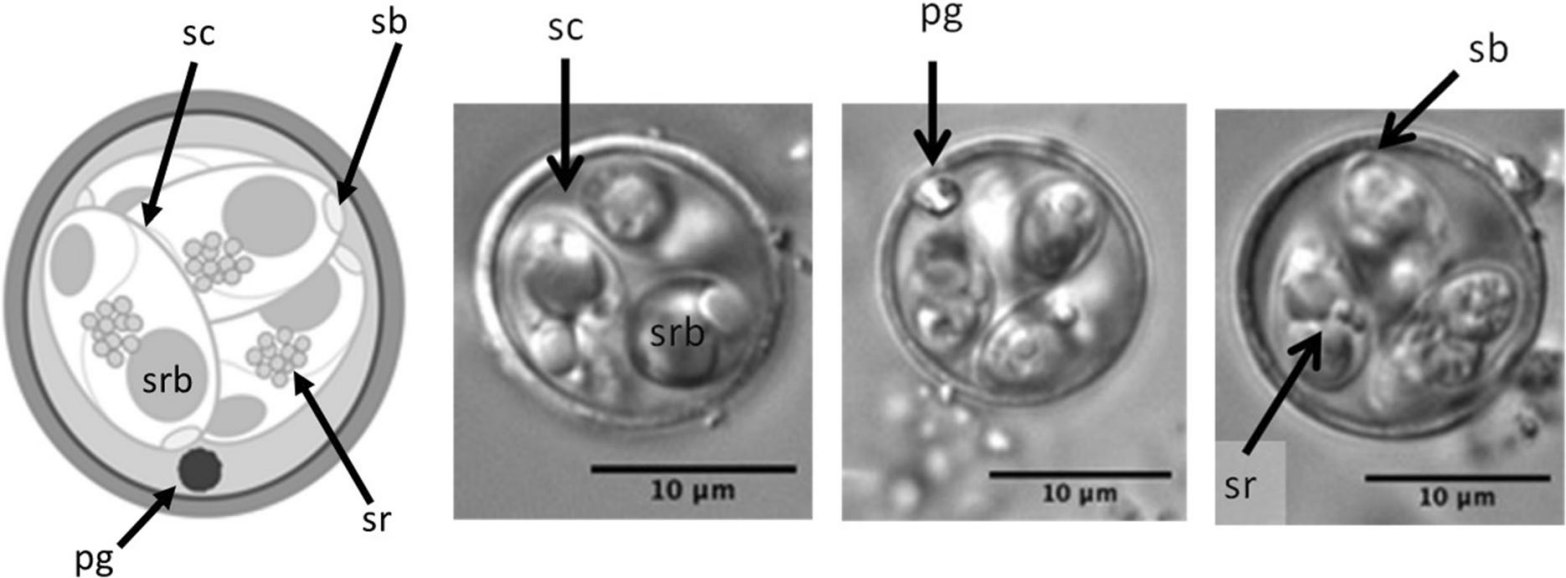
Line drawing and photomicrograph of the novel morphotype (*E. paopaoii*, n=84) reported in brown kiwi (*Apteryx mantelli*). Key: sc = sporocyst, srb = sporozoite anterior refractile body, pg= polar granule, sr = sporocyst residuum, sb = Stieda body

**Fig. 2:**
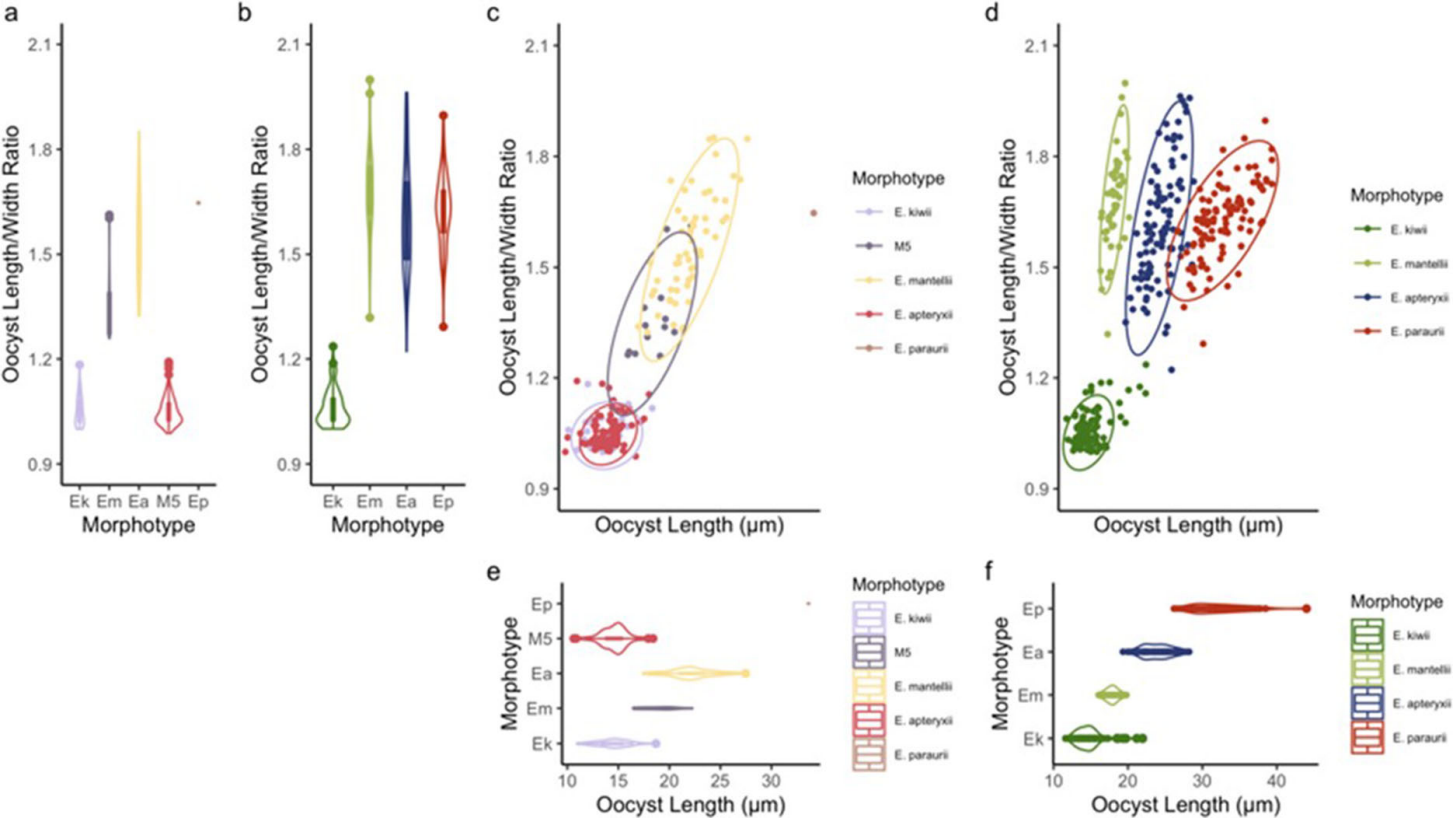
Six plots comparing coccidia from brown kiwi (*Apteryx mantelli*) from this study and Morgan et al. (2017). Plots “a”, “c” and “e” are from this study whereas plots “b”, “d” and “f” are from Morgan et al. (2017). Plots “a” and “b” are violin (density) plots comparing the oocyst length/width ratios. Plots “c” and “d” are scatterplots sorted by oocyst length and length/width ratio with data. Plots “e” and “f” are violin (density) plots comparing the oocyst length of morphotypes. Morphotypes *E. kiwii* and *E. paopaoii* (M5) are distinguished by a striated and smooth wall, respectively. *E. mantellii* and *E. apteryxii* are distinguished by size and the absence and presence of a visible micropyle, respectively

Of the oocysts characterised for this study, 84 morphologically similar oocysts, named *Eimeria paopaoii* (Fig. 1) for the purpose of this study, did not fit the descriptions of the species reported by Morgan et al. (2017). While similar to *E*. *kiwii*, this novel fifth morphotype, *E. paopaoii*, has a smooth rather than striated wall. This qualitative distinction was reliably discernible under oil immersion and occasionally discernible at lower magnifications. In addition to this novel morphotype, the measurements of *E. mantellii* and *E. apteryxii* taken in the present study showed these morphotypes to be less distinguished by size than previously described (Fig. 2; Table 4). The average dimensions of *E. mantellii* were reported to be 17.9 x 10.7 $\mu$ m with a 1.7 length/width (L/W) ratio (Morgan et al., 2017), whereas the current research reports a mean of 18.8 x 13.9 $\mu$ m and a L/W ratio of 1.4. While the sizes of these two reports are relatively similar, the L/W ratio provides a better metric for shape and indicates an overall difference. This inconsistency is illustrated in the increasing overlap in L/W ratio between *E. mantellii* and *E. apteryxii* (Fig. 2c).

**Table 4:** Comparison of the morphometrics of kiwi *Eimeria* in the present study (A) and those reported by Morgan et al. (2017) (B). Values not reported in Morgan et al. (2017) were retrieved from Morgan (2013)

		Oo. Length x Width (avg) $\mu$ m	Oo. L/W (avg)	Sporocyst Length (avg) $\mu$ m	Sporocyst L/W (avg)	Wall Width (avg) $\mu$ m	No. PG (avg)	PG Size (avg) $\mu$ m	Micropyle (avg)$\mu$ m
***E. kiwii***	A (n = 34)	10.6-18.7 x 12.7-16.7 (16.7 x 13.7)	1.0-1.2 (1.1)	6.7-12.0 x 4.1-6.4 (9.1 x 5.0)	1.4-2.3 (1.8)	0.6-1.0 (0.72)	1	1.1-2.8 x 0.9-2.4 (2.1 x 1.6)	Absent
	B (n = 100)	11.6-22.0 x 10.6-19.0 (14.8 x 13.9)	1.0-1.2 (1.1)	6.5–13.6 x 3.6–7.4 (9.4 x 4.9)	1.5-2.3 (1.9)	0.5-1.2 (0.79)	1-2	1.5-2.0 × 1.0-2.1 (2.1 × 1.6)	Absent
***E. mantellii***	A (n = 13)	16.4-22.3 x 12.5-15.7 (18.9 x 13.9)	1.3-1.6 (1.4)	7.8-12.5 x 4.7-6.7 (10.9 x 5.7)	1.4-2.2 (1.9)	0.5-0.9 (0.7)	1-5 (2.667)	0.6-2.4 x 0.6-2.2 (1.5 x 1.1)	Absent
	B (n = 50)	16.1-19.8 x 9.6-12.9 (17.9 x 10.7)	1.4-2.0 (1.7)	7.9–10.0 x 3.9–5.2 (9.2 x 4.5)	1.6-2.4 (2.0)	0.4-0.8 (0.62)	1-2	0.9-2.1 × 0.4-1.6 (1.5 × 1.1)	Absent
***E. apteryxii***	A (n = 59)	19.2-27.5 x 12.7-16.1 (22.2 x 14.4)	1.3-1.9 (1.6)	8.5-13.6 x 3.8-9.0 (11.4 x 5.9)	1.2-3.3 (2.0)	0.4- 0.9 (0.70)	1-7 (2.900)	0.6-3.2 x 0.5-2.4 (1.6 x 1.2)	1.1-3.2 (2.0)
	B (n = 100)	19.3-28.2 x 12.7-20.7 (23.9 x 14.9)	1.2-2.0 (1.6)	8.0–17.4 x 5.0–8.3 (11.7 x 6.0)	1.4-2.4 (1.9)	0.6-1.2 (0.8)	1-7	1.2-2.9 × 0.8-2.1 (2.2 × 1.3)	1.6-2.4 (2.0)
***E. paraurii***	A (n = 1)	33.6 x 20.5 (N/A)	1.7 (N/A)	17.8 x 8.0 (N/A)	2.2 (N/A)	1.1 (N/A)	1 (N/A)	3.2 x 2.8 (N/A)	3.1 (N/A)
	B (n = 100)	26.2-44.0 x 16.4-23.0 (32.2 x 19.8)	1.3-2.8 (1.6)	11.7-20.6 x 6.8–9.2 (16.2 x 7.9)	1.5-2.3 (2.0)	0.8-1.7 (1.2)	1-2	Not measured	Absent
***E. paopaoii***	A (n = 84)	10.6-18.4 x 9.8-17.2 (14.6 x 13.9)	1.0-1.2 (1.1)	5.8-11.5 x 3.6-6.8 (9.4 x 5.1)	1.4-2.2 (1.9) n=106	0.5-0.9 (0.7)	1	1.1 -3.3 x 0.9-2.4 (2.3 x 1.8)	Absent

.

**Morphological descriptions**

***Family:*** Eimeriidae (Minchin 1903)

***Genus:**** Eimeria* (Schneider 1875)

***Isolate name:**** Eimeria paopaoii* – (Fig. 1)

***Host:*** Brown kiwi, *Apteryx mantelli* (Bartlett 1852; Burbidge et al. 2003), Juvenile.

***Localities:*** Pūkaha National Wildlife Centre, Wairarapa, New Zealand (40°43′35.0″S 175°38′23.6″E); Warrenheip, Waikato, New Zealand (37°56′19.7″S 175°43′08.5″E); and The National Kiwi Hatchery Aotearoa, Rotorua, New Zealand (38°06′33.5″S 176°13′14.3″E).

***Deposited material:*** Photomicrographs and oocysts in K_2_Cr_2_O_7_ and frozen at -80°C are deposited in the Massey University Parasitology Collection under the reference: *E. paopoaii* (morphotype 5) 2018/1

***Prevalence:*** 86% (in 6 of 7 specimens).

***Sporulation time:*** Exogenous. All oocysts were passed unsporulated and sporulated within 15 days at room temperature.

***Site of infection:*** Unknown; retrieved from droppings.

***Sporulated oocyst:*** Oocyst shape (n = 84) circular to elliptic: 10.6-18.4 $\mu$ m) $\times$ 9.8-17.2 $\mu$ m (14.6 $\times$ 13.9 $\mu$ m; length/width (L/W) ratio 1.0-1.2 (avg 1.1). Smooth wall, 0.5-0.9 $\mu$ m) (avg 0.7). Micropyle, micropylar cap, and oocyst residuum absent. 1 polar granule present.

***Sporocyst:*** Sporocysts (n = 106), 5.8-11.5 $\mu$ m $\times$ 3.6-6.7 $\mu$ m (9.4 $\times$ 5.1 $\mu$ m); length/width (L/W) ratio 1.3-2.2 (avg 1.9); stieda body present; sporocyst residuum present, generally clumped.

***Sporozoite:*** Two sporozoites, not measured; large sporozoite refractile body at the posterior end.

***Etymology****:* Paopao is the Te Reo Maori verb “to hatch”, this isolate was found at Operation Nest Egg National Hatchery locations which collect eggs from the wild, hatch and care for kiwi chicks until they can be safely returned to the wild. Te Reo Māori is the language of the indigenous people of New Zealand.

***Taxonomic remarks:*** This is similar to *E. kiwii* described by Morgan et al. (2017); however, this species is characterised by a smooth wall rather than a striated wall*.*

***Molecular analysis***

*COI gene*

**Fig. 3:**
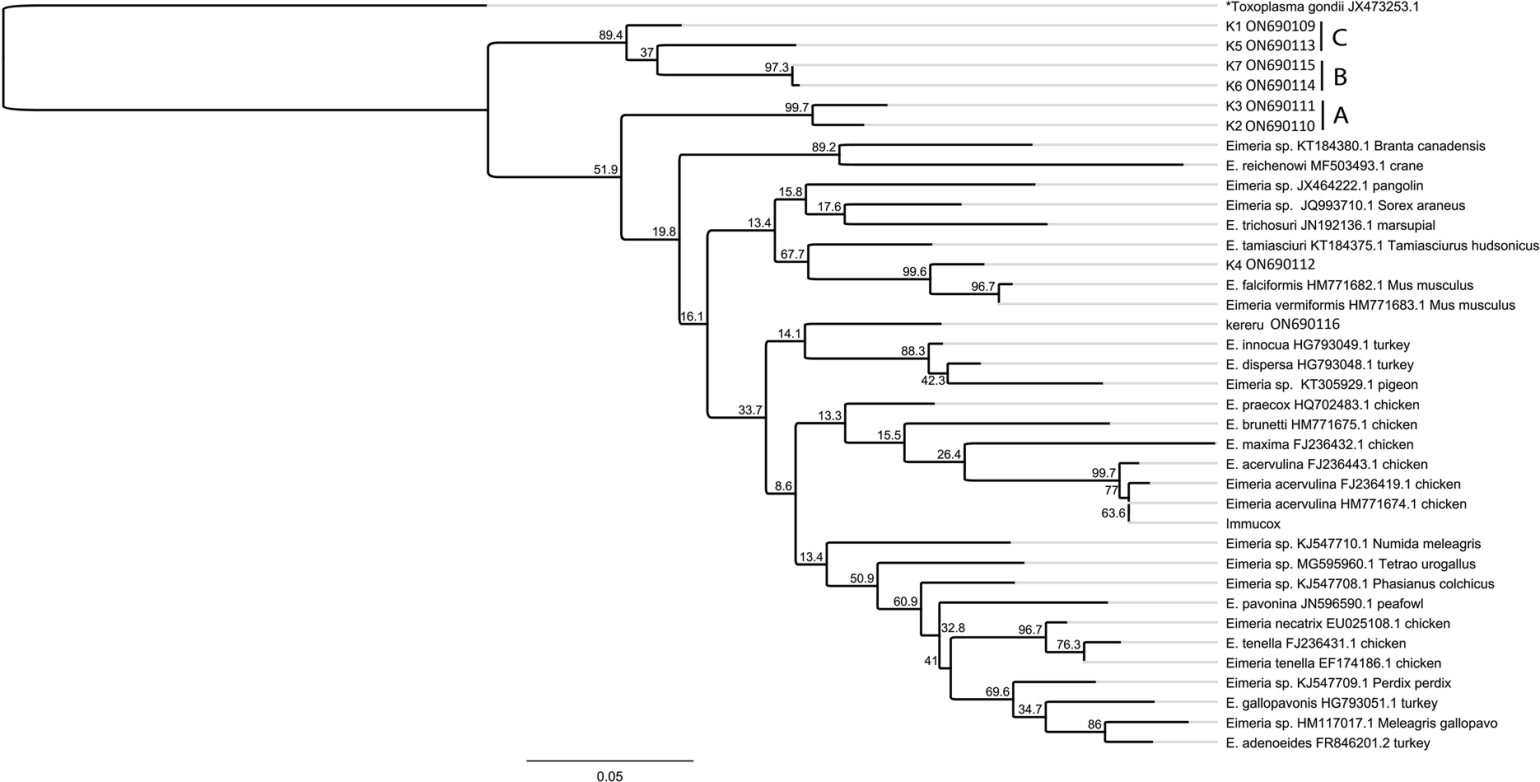
Maximum likelihood consensus tree of kiwi coccidia at the mitochondrial cytochrome *c* oxidase I gene (459 base pairs) with *Toxoplasma gondii* as the outgroup. Tamora-Nei, 93 distance model with 1000 replicates was used to calculate branch length (scale); bootstrap values range from 8.6 to 100

The COI protocol provided sufficient amplification to yield high-quality chromatograms although overlapping peaks, indicative of co-infection with multiple *Eimeria* spp., were present in three of the seven samples and the Immucox® Breeders and Layers control. The novel kiwi *Eimeria* isolates (K1-7) have been submitted to the NCBI database (Accession# ON690109-ON690115, respectively). Consensus sequences confirmed that the sequences generated from the coccidia from all seven samples and the Immucox® Breeders and Layers control fell within the genus *Eimeria* (Fig. 3).

However, the sequence obtained from sample K4, which was collected from Orana Wildlife Park in Christchurch, was 99% similar to a mouse coccidia sequence *Eimeria ferrisi* Levine & Ivens 1965 (GenBank MH777579.1) and grouped with another species of mouse coccidia, *Eimeria falciformis* (Eimer 1870; GenBank HM771682.1). Thus, with the exception of K4, which was likely environmental contamination, the remaining six kiwi coccidia sequences fell into three main clusters, which have been grouped into clusters A-C (Fig. 3). This clustering includes the ambiguous bases, ranging from 1 (0.2%) to 16 (3.5%), that occur in four of these sequences.

Cluster A contained K2 and K3, which had a nucleotide sequence identity of 95%. The samples in this cluster originated from the Manawatu region. Cluster B contained K6 and K7, which shared 98% identity; both originated from the Waikato region. Cluster C, which contained K1 and K5, was the most divergent; these nucleotide sequences were no more than 93% identical with each other or any other sample from this study.

With the exception of the K4, the kiwi samples did not share strong nucleotide sequence identity with records documented in GenBank at this COI site. The most similar nucleotide sequence was from K2, which was 91% identical with an *Eimeria mundayi* Barker, O'Callaghan & Beveridge 1988 (GenBank MK202808.1) from a woylie (*Bettongia penicillata* Gray) in Australia.

## Discussion

***Morphology***

In this study, five *Eimeria* morphotypes were reported from the seven pooled faecal samples from brown kiwi with one novel morphotype (*E. paopaoii*) described. It should be noted that there is some size and shape variation of several *Eimeria* species reported in this study compared with the previously described examples by Morgan et al. (2017). It is likely this variation is due to the limited number of oocysts measured (1, 13, or 34 oocysts) in this study compared to the larger sample size (100, 50, or 100 oocysts respectively) presented by Morgan et al (2017). It is expected that as the number of oocysts identified and measured for each species increases, the degree of error will decrease, improving morphological identification; thus, the addition of this data should be considered a continuation of the characterisations of these species as the differences may reflect natural variation. Furthermore, the main distinction between examples of these morphotypes from the present report relies heavily on the absence/presence, respectively, of a visible micropyle. The possibility remains that the present or previous study relies on technology (such as imaging software) that increases or decreases the likelihood of micropyles remaining unobserved. Differences in oocyst isolation for imaging may also have had an impact on characterisation. For example, the integrity of oocysts may be affected by the osmotic pressure of the flotation solution, an effect that increases the longer the oocysts are exposed (Ballweber et al., 2014; Zajac and Conboy, 2012).

Similarly, although the same methods were used by Morgan et al. (2017) as the present study, the addition of the *E. paopaoii* morphotype in this report could reflect operator variation, rather than the absence of this morphotype in the previous dataset. Nevertheless, the clear differences between the smooth and rough walls confirm the presence of at least five morphotypes in brown kiwi.

Interestingly, only one *E. paraurii* oocyst was identified in these samples, whereas it was identified in all (6/6) samples examined by (Morgan et al., 2017). *E. mantellii* was present in 71% (5/7) of samples examined in this study and only 17% (1/6) previously. Alternatively, the prevalence of *E. apteryxii* and *E. kiwii* were similar (86% and 100%, respectively) between these reports (Morgan et al., 2017). This variation in prevalence indicates the species composition of coccidia infecting kiwi probably varies over time and location.

***Molecular analysis***

The differentiation provided by the COI gene confirms the presence of novel species of *Eimeria* in brown kiwi. The sequences grouped in clusters A-C (Fig. 3) potentially representing two or more of the morphospecies reported here. Unfortunately, Sanger sequencing tends to lead to the detection of only the most common sequence (Altimari et al., 2013; Cereb et al., 2015; Magalhaes et al., 2015) which was highlighed by the Immucox® Breeders and Layers PCR control aligning mostly closely with *E. acervulina* despite containing four distinct *Eimeria* species (Fig. 3). However, combining the morphology data (Table 3) with the percent identities of the most common sequence may enable predictions to be made between a morphotype and a particular sequence. For example, the samples in clade A, K2 and K3, were the most similar with a 98% identity. Sample K2 contained predominately *E. apteryxii* (74%, 35/47 oocysts), whereas sample K3 only contained 25% (1/4 oocysts) of *E. apteryxii.* Thus, the K2 sequence may represent *E. apteryxii*, as it contained only a single ambiguous base. On the other hand, the K3 sequence had no ambiguous bases and contained three morphotypes. Sanger sequencing has been shown to preferentially yield the most dominant sequence, leading to missed variation (Altimari et al., 2013; Cereb et al., 2015; Magalhaes et al., 2015). This tendency is further illustrated by the K4 sample, which yielded an unambiguous sequence from mouse coccidia even though microscopically, the sample contained four kiwi *Eimeria* morphotypes (Table 3). The oocyst length of *Eimeria ferrisi* from mice ranges from 12-22 $\mu$ m (17 $\mu$ m) with a width ranging from 11-18 $\mu$ m (Ankrom et al., 1975). The length to width ratio of this species is 1.22 (1 to 1.6) and spherical to ovoid with a smooth wall. The sporocysts of *E. ferrisi* measure 8-11 $\mu$ m (17 $\mu$ m) in length and 5-7 $\mu$ m (5.9 $\mu$ m) in width. This description most resembled *E. paopaoii* oocysts, however, while the oocyst and sporocyst sizes were similar, the L/W ratio of the *E. paopaoii* morphotype ranged from 1.00-1.19 (1.05). The difference in oocyst L/W ratio as well as the description of *E. paopaoii* in other kiwi faecal samples suggests that these oocysts were properly morphologically identified as *E. paopaoii*. Interestingly, the COI gene sequence from this sample was clean with no overlapping peaks. This preferential sequencing of a non-target species of *Eimeria* demonstrates the importance of using several methods for the identification of oocysts in novel hosts.

Half (n = 3) of the samples successfully sequenced for the COI gene target contained many ambiguous regions, supporting the presence of co-infection. The similarity (88.9%) between the sequences enabled enough variation between the samples to provide meaningful comparisons between species of kiwi coccidia in brown kiwi, while remaining conserved enough for further diagnostic testing development and use in comparison to other *Eimeria*. The small sample size and the number of ambiguities in the COI sequencing results encourages further research into the genetic variation of these kiwi *Eimeria*. Increasing the sample size and range would encourage a greater representation of the diversity of coccidia infecting brown kiwi. A deeper sequencing analysis (e.g., Next Generation Sequencing) is encouraged to produce a truer representation of the variation within and between samples, especially as producing a guaranteed, single-species infection in naturally infected kiwi is unlikely.

In the absence of experimentally infected kiwi, the best replacement would be single oocyst sequencing, which allows for definitive connections between morphotypes and particular sequences. However, extracting DNA from a single oocyst can be unreliable (Morgan, 2013). Amplifying the DNA from a single oocyst has been approached in several ways. The first relies on the dilution of a high concentration of oocysts to the equivalent dilution of a single oocyst (Molloy et al., 1998). Alternatively, the dilution of DNA (e.g., *Eimeria* spp. in chickens; Vrba et al., 2010) to the concentration of a single haploid *Eimeria* spp. cell can also be used; however, this method may be more useful for testing the reliability of a particular amplification assay, rather than the reliable amplification of a single oocyst. Unfortunately, this dilution method is imprecise; with each dilution, the errors associated with manipulating small volumes increases, leading to progressively poorer estimates of true concentrations (Grgicak et al., 2010). Thus, physical visualisation and manipulation of individual oocysts provides a potential solution. The main tools that have allowed for the development of this method is micropipettes and micromanipulators. For example, Dolnik et al. (2009) used microscopic confirmation and micropipettes to isolate individual *Isospora* spp (Schneider 1881) oocysts for amplification. This resulted in 72% (n = 39) successful, unambiguous sequences. Similarly, Sturbaum et al. (2001) described a method using a micropipette, micromanipulator, as well as microscopic confirmation to isolate *Cryptosporidium parvum* (Tyzzer 1912), which have oocysts up to 6 $\mu$ m in diameter, with an 89% success rate. The lower success rate of detection and unknown reliability of the selected primers to amplify *Eimeria* endemic to New Zealand led the authors to pursue the more reliable form of extraction (i.e., many oocysts at a time). This removed controllable factors of uncertainty. For example, even if a single oocyst is isolated from a sample, the possibility remains that liquid in the sample contained DNA from another, morphologically distinct oocyst. Thus, as an initial description, the authors believed it more important to establish the ability to detect these *Eimeria* and to provide baseline molecular references for future comparison.

***Management implications***

Future morphological research should focus on describing coccidia in other species of kiwi. For example, Haast tokoeka (*A. australis* “Haast”, Shaw 1813) also rely heavily on ONE for continued population growth and are commonly raised in facilities that house brown kiwi on site. Determining whether these species of coccidia also infect other kiwi species would determine the extent to which these parasites need to be managed. The host specificity of kiwi coccidia is currently unknown.

Additionally, as Okarito kiwi (rowi, *Apteryx rowi* Tennyson et al.) are most closely related to brown kiwi (Weir et al., 2016), *Eimeria* species that can parasitise brown kiwi are most likely to be capable of reproducing in rowi. There are a number of examples where a species of *Eimeria* is capable of infecting closely related host species (e.g., *Eimeria gruis* Forrester et al. 1978 and *Eimeria reichenowi* Courtney et al. 1975 in hooded cranes *Grus monacha* Temminck, white-naped cranes *Antigone vipio* (Pallas), sandhill cranes *Antigone canadensis* (Linnaeus), and whooping cranes *Grus americana* (Linnaeus) (Honma et al., 2011a; Honma et al., 2011b; Novilla et al., 1981). While the last known land bridge between the North and South Island occurred only 20,000 years ago, the glacial period drove rapid diversification of kiwi through geographic isolation and bottlenecking (Weir et al., 2016). During periods of geographic isolation and diversification, it would be expected for the coccidia of kiwi to also diversify. Whether or not some (or all) of these parasites differentiated enough to become specific to a particular species of kiwi has yet to be determined.

***Conclusions***

This study provides insight into the genetic variation of coccidia in kiwi and demonstrates the complexity of studying coccidia in kiwi. The samples included in this study are all from a small number of kiwi in captivity or crèches and, therefore, likely do not reflect the variation found throughout New Zealand. Identifying coccidia in wild populations of high conservation value would ensure novel parasites were not introduced into naïve populations through the release of captive-reared kiwi potentially carrying coccidia. Further, ONE facilities frequently house multiple species of kiwi, and knowledge of host-specificity will identify the risk of exposure of kiwi to novel, highly pathogenic species of *Eimeria* via cross contamination of enclosures. This introduction to naïve populations could not only lead to decreased reproductive success of the kiwi but could also lead to the introduction of an invasive parasite that could outcompete the endemic *Eimeria*. Therefore, characterisation of coccidia in other kiwi species should be a priority. Additionally, the development of rapid, non-invasive, highly specific tools to monitor treatment efficacy is vital to ensuring ONE uses resources wisely. Such a tool needs to account for as much of the variation in kiwi coccidia as possible to ensure rare, but highly virulent species are not overlooked. This variation is likely to be missed unless more in-depth sequencing technologies are used.

## Data Availability

Sequences have been deposited into NCDI GenBank https://www.ncbi.nlm.nih.gov/ (Accession numbers ON690109-ON690115).

## References

[CR1] Altimari A, de Biase D, De Maglio G, Gruppioni E, Capizzi E, Degiovanni A, D’Errico A, Pession A, Pizzolitto S, Fiorentino M (2013). 454 next generation-sequencing outperforms allele-specific PCR, Sanger sequencing, and pyrosequencing for routine KRAS mutation analysis of formalin-fixed, paraffin-embedded samples. Oncol Targets and Therapy.

[CR2] Ankrom SL, Chobotar B, Ernst JV (1975). Life Cycle of *Eimeria ferrisi* Levine & Ivens, 1965 in the Mouse, *Mus musculus*. Journal of Protozoology.

[CR3] BirdLife International. 2017. *Apteryx mantelli* (2017) 10.2305/IUCN.UK.2017-3.RLTS.T45353580A119177586.en. Accessed 27 February 2018.

[CR4] Ballweber LR, Beugnet F, Marchiond AA, Payne PA (2014). American Association of Veterinary Parasitologists' review of veterinary fecal flotation methods and factors influencing their accuracy and use–is there really one best technique?. Veterinary Parasitology.

[CR5] Burbidge ML, Colbourne RM, Robertson HA, Baker AJ (2003). Molecular and other biological evidence supports the recognition of at least three species of brown kiwi. Conservation Genetics.

[CR6] Cereb N, Kim HR, Ryu J, Yang SY (2015). Advances in DNA sequencing technologies for high resolution HLA typing. Humun Immunology.

[CR7] Coker SM, Pomroy WE, Howe L, McInnes K, Vallee E, Morgan KJ (2020). Comparing the Mini-FLOTAC and centrifugal faecal floation for the detection of coccidia (*Eimeria* spp.) in kiwi (*Apteryx mantelli*). Parasitology Research.

[CR8] Colbourne R (2005). The development of Operation Nest Egg as a tool in the conservation management of kiwi. Science for Conservation.

[CR9] da Silva-Carvalho LM, Pastura DGN, Gomes JV, Siqueria PB, Rodrigues MB, de Lima VM, Berto BP (2018). *Isospora lopesi* n. sp. (Apicomplexa: Eimeriidae) from the eastern white-throated spadebill *Platyrinchus mystaceus* Vieillot (Passeriformes: Tyranni: Tyrannidae) in South America. Systematic Parasitology.

[CR10] da Silva-Carvalho LM, Genovez-Oliveira JL, de Souza Oliveira M, de Oliveria JL, de Lima VM, Ferreira I, Berto BP (2020). Polymorphism and genetic diversity of *Isospora parnaitatiaiensis* Silva, Rodrigues, Lopes, Berto, Luz, Ferreira & Lopes, 2015 (Eimeriidae) from antbirds (Thamnophilidae) in Brazil. Systematic Parasitology.

[CR11] Dolnik OV, Palinauskas V, Bensch S (2009). Individual oocysts of *Isospora* (Apicomplexa: Coccidia) parasites from avian feces: from photo to sequence. Journal of Parasitology.

[CR12] Duszynski DW, Wilber PG (1997). A guideline for the preparation of species descriptions in the Eimeriidae. Journal of Parasitology.

[CR13] Fernandez-Alvarez A, Modry D, Foronda P (2016). A new species of *Eimeria* Schneider, 1875 (Apicomplexa: Eimeriidae) from *Alectoris barbara* (Aves: Phasianidae) from the Canary Islands (Spain). Parasitololgy Research.

[CR14] Grgicak CM, Urban ZM, Cotton RW (2010). Investigation of reproducibility and error associated with qPCR methods using Quantifiler® Duo DNA quantification kit. Journal of Forensic Sciences.

[CR15] Honma, H., Suyama, Y., Nakai, Y. (2011a) Detection of parasitizing coccidia and determination of host crane species, sex and genotype by faecal DNA analysis. *Molecular Ecology Resourses*, 11,:1033–1044. 10.1111/j.1755-0998.2011.03048.x 10.1111/j.1755-0998.2011.03048.x 21791031

[CR16] Honma, H., Suyama, Y., Watanabe, Y., Matsumoto, F., Nakai, Y. (2011b) Accurate analysis of prevalence of coccidiosis in individually identified wild cranes in inhabiting and migrating populations in Japan. *Environmental Microbiology*, 13, 2876–2887. 10.1111/j.1462-2920.2011.02563.x 10.1111/j.1462-2920.2011.02563.x 21895916

[CR17] Jenkins MC, Dubey JP, Miska K, Fetterer R (2017). Differences in fecundity of *Eimeria maxima* strains exhibiting different levels of pathogenicity in its avian host. Veterinary Parasitology.

[CR18] Magalhaes S, Marques SL, Alves C, Amorim A, Alvarez L, Goios A (2015). Evaluation of heteroplasmy detection in the Ion Torrent PGM. Forensic Science International Genetics Supplement Series.

[CR19] Maronezi C, Oliveira MD, Genovez-Oliveira JL, de Mello ER, Cepeda PB, de Oliveria AA, de Lima VM, Berto BP (2022). *Isospora* spp. (Eimeriidae) from green-winged saltators *Saltator similis* d'Orbigny & Lafresnaye, 1837 (Thraupidae) from captivity near the conservation unit of the Itatiaia National Park in Southeastern Brazil. Systematic Parasitology.

[CR20] McAllister CT, Hnida JA, Woodyard ET, Rosser TG (2019). *Eimeria* spp. (Apicomplexa: Eimeriidae) from great horned owls, *Bubo virginianus* (Gmelin) (Aves: Strigiformes) from Arkansas and Oklahoma, USA, with novel molecular information on *Eimeria bubonis* Cawthorn & Stockdale, 1981. Systematic Parasitology.

[CR21] McAllister CT, Hnida JA (2020). A new species of *Eimeria* (Apicomplexa: Eimeriidae) from eastern woodrat, *Neotoma floridana* (Rodentia: Cricetidae), from Arkansas, USA, and a summation of eimerians from North American woodrats. Acta Parasitologica.

[CR22] McLennan JA, Dew L, Miles J, Gillingham N, Waiwai R (2004). Size matters: predation risk and juvenile growth in North Island brown kiwi (Apteryx mantelli). New Zealand Journal of Ecology.

[CR23] McLennan, J. A., et al. (1996) Role of predation in the decline of kiwi, Apteryx spp., in New Zealand. *New Zealand Journal of Ecology*, 20(1), 27–35.

[CR24] Medina, J. P., Medina-Valdez, H., Sánchez-Jasso, J. M., García-Albarrán, M., Salgado-Miranda, C., Soriano-Vargas, E. (2019) *Eimeria aegoliusia* n. sp. (Sporozoa: Eimeriidae) from the northern saw-whet owl *Aegolius acadicus* (Gmelin) (Strigiformes: Strigidae) in Mexico. *Systematic Parasitology*, 96(6), 521–526. 10.1007/s11230-019-09863-x 10.1007/s11230-019-09863-x 31089939

[CR25] Molloy JB, Eaves FW, Jeston PJ, Minchin CM, Stewart NP, Lew AE, Jorgensen WK (1998). Detection of *Eimeria acervulina* using the polymerase chain reaction. Avian Diseases.

[CR26] Morgan JAT, Godwin RM (2017). Mitochondrial genomes of Australian chicken *Eimeria* support the presence of ten species with low genetic diversity among strains. Veterinary Parasitology.

[CR27] Morgan, K. (2013) Coccidiosis in the kiwi (*Apteryx* spp): Aspects of the pathology, epidemiology and parasite biology. Dissertation, Massey University, Palmerston North, New Zealand.

[CR28] Morgan KJ, Alley MR, Pomroy WE, Castro I, Howe L (2012). Enteric coccidiosis in the brown kiwi (*Apteryx mantelli*). Parasitololgy Research.

[CR29] Morgan KJ, Alley MR, Pomroy WE, Gartrell BD, Castro I, Howe L (2013). Extra-intestinal coccidiosis in the kiwi (*Apteryx* spp.). Avian Pathology.

[CR30] Morgan KJ, Castro I, Lopez-Villalobos N, Pomroy WE, Alley MR, Gartrell BD, Hunter S, Howe L (2014). Prevalence of and risk factors for coccidiosis in kiwi between 1977 and 2011. New Zealand Veterinary Journal.

[CR31] Morgan KJ, Pomroy WE, Howe L, Alley MR, Castro I (2017). Description of four new species of coccidia (Apicomplexa: Eimeriidae) from brown kiwi, *Apteryx mantelli*, in New Zealand. Parasitology Research.

[CR32] Novilla MN, Carpenter JW, Spraker TR, Jeffers TK (1981). Parental Development of Eimerian Coccidia in Sandhill and Whopping Cranes. Journal of Protozoology.

[CR33] Ogedengbe JD, Hunter DB, Barta JR (2011). Molecular identification of *Eimeria* species infecting market-age meat chickens in commercial flocks in Ontario. Veterinary Parasitology.

[CR34] Robertson, H., Baird, K., Dowding, J. E., Elliot, G. P., Hitchmough, R. A., Miskelly, C. M., McArthur, N., O’Donnell, C. F. J., Sagar, P. M., Scofield, R. P., Taylor, G. A. (2017) Conservation status of New Zealand birds, 2016. Wellington: Conservation, D.o., ed.

[CR35] Schneider CA, Rasband WS, Eliceiri KW (2012). NIH Image to ImageJ: 25 years of image analysis. Nature Methods.

[CR36] Sturbaum GD, Reed C, Hoover PJ, Jost BH, Marshall MM, Sterling CR (2001). Species-specific, nested PCR-restriction fragment length polymorphism detection of single *Cryptosporidium parvum* oocysts. Applied Environmental Microbiology.

[CR37] Taylor HS, Morgan KJ, Pomroy WE, McInnes K (2019). Apparent lack of efficacy of toltrazuril against *Eimeria* species affecting brown kiwi (*Apteryx mantelli*) at a captive rearing facility. New Zealand Veterinary Journal.

[CR38] Thompson EJ, Wright IG (1978). Coccidiosis in kiwis. New Zealand Veterinary Journal.

[CR39] Thompson JD, Higgins DG, Gibson TJ (1994). CLUSTAL W: improving the sensitivity of progressive multiple sequence alignment through sequence weighting, position-specific gap penalties and weight matrix choice. Nucleic Acids Research.

[CR40] Vrba V, Blake DP, Poplstein M (2010). Quantitative real-time PCR assays for detection and quantification of all seven *Eimeria* species that infect the chicken. Veterinary Parasitology.

[CR41] Weir JT, Haddrath O, Robertson HA, Colbourne RM, Baker AJ (2016). Explosive ice age diversification of kiwi. Procedings of the National Academy of Sciences USA.

[CR42] Williams RB (2001). Quantification of the crowding effect during infections with the seven Eimeria species of the domesticated fowl: its importance for experimental designs and the production of oocyst stocks. International Journal for Parasitology.

[CR43] Woodyard ET, Rush SA, Rosser TG (2019). Redescription of *Eimeria megabubonis* Upton, Campbell, Weigel & McKown, 1990 (Apicomplexa: Emeriidae) from the great horned owl *Bubo virginianus* (Gmelin). Systematic Parasitology.

[CR44] Yabsley, M. J. (2008) Eimeria. In: Atkinson CT, Thomas NJ, Hunter DB (eds) *Parasitic Diseases of Wild Birds*. Wiley-Blackwell, Ames, p 162–180.

[CR45] Yang R, Brice B, Bennett MD, Eliott A, Ryan U (2013). Novel *Eimeria* sp. isolated from a King's skink (*Egernia kingii*) in Western Australia. Experimental Parasitology.

[CR46] Zajac AM, Conboy GA (2012). Veterinary clinical parasitology.

